# Relationship between Perioperative Cardiovascular Events and Glycated Hemoglobin in Diabetic Patients Undergoing Noncardiac Surgery

**DOI:** 10.1155/2020/3645374

**Published:** 2020-12-17

**Authors:** Zhengwen Chen, Shuncai Ding, Yingchuan Yuan, Jianhua Du, Ling Zhang

**Affiliations:** ^1^Department of Anesthesia, The Second Affiliated Hospital to Xinjiang Medical University, Urumqi 830000, China; ^2^Department of Cadre Health Care, People's Hospital of Xinjiang Uygur Autonomous Region, Urumqi 830001, China

## Abstract

This retrospective nested case-control study is aimed at investigating the relationship between HbAlc and perioperative cardiovascular events (PCE) in patients with diabetes who underwent complex or moderately complex noncardiac surgery at the Second Affiliated Hospital of Xinjiang Medical University in 2013-2018. The patients were divided into four groups according to HbA1c ≤7%, 7.1%-7.9%, 8.0%-8.9%, and ≥9%. The occurrence of PCE among the groups was compared using univariable and multivariable analyses. Finally, 318 patients were included. There were 90 cases of PCE among the 318 patients; the occurrence rate of PCE was 28.3%. No death occurred. The occurrence rates of PCE in the HbA1c ≥ 8.0% − 8.9% and HbA1c ≥ 9.0% groups were 30.8% and 35.4%, respectively (*P* < 0.001 vs. the HbA1c 7.1%-7.9% group). The occurrence rate of PCE in the HbA1c ≤ 7% group was 25.9% (*P* > 0.05 vs. the HbA1c 7.1%-7.9% group). The multivariable logistic regression analysis showed that the course of diabetes (HbA1c stratification ≤7%, 7.1%-7.9%, 8.0%-8.9%, ≥9%, OR = 3.672, 95% CI: 1.552-8.687), HbA1c (OR = 1.895, 95% CI: 1.227-4.830), SBP (OR = 1.194, 95% CI: 1.015-2.023), and microalbuminuria (OR = 1.098, 95% CI: 1.005-1.023) was independently associated with PCE in diabetic patients. In conclusion, HbA1c levels are related to the incidence of PCE in diabetic patients undergoing complex or moderately complex noncardiac surgery.

## 1. Introduction

Diabetes mellitus type 2 (T2DM) is a common endocrine disorder characterized by variable degrees of insulin resistance and deficiency, resulting in hyperglycemia [1]. The worldwide prevalence of T2DM was 9.0% in men and 7.9% in women in 2014 [2] and 8.5% in the United States in 2016-2017 [3]. The potential complications of T2DM include cardiovascular disease, neuropathy, nephropathy, retinopathy, and increased mortality [4]. T2DM is often identified through routine screening beginning in middle age or through targeted screening of adults of any age with overweight or obesity and with risk factors such as metabolic syndrome, polycystic ovary syndrome, a history of gestational diabetes, or other concerning familial, clinical, or demographic characteristics [1].

Blood glucose levels represent the short-term glucose burden, while the glycated hemoglobin (HbA1c) levels represent the long-term glycemic burden [1]. HbA1c target of <7% is considered a reasonable target in adults [1]. The usual management of diabetes includes lifestyle modifications, weight loss, and hypoglycemic agents substitute for lipid-lowing medications such as metformin, sulfonylurea, thiazolidinedione, DDP-4 inhibitors, SGLT2 inhibitor, and GLP-1 agonists [1, 5].

Diabetes has been recognized as an independent risk factor for perioperative cardiovascular events (PCE) in patients undergoing cardiac or noncardiac surgery [6, 7]. Nevertheless, at present, there are only a few reports with inconsistent conclusions about the effect of blood glucose control on the risk of PCE, and previous studies did not perform a detailed stratification of blood glucose control level or further analysis of the traditional cardiovascular risk factors [8, 9]. HbA1c is a good indicator to evaluate the blood glucose level of diabetic patients [1]. Nevertheless, previous studies report conflicting results about the association between HbA1c levels and PCE, with studies reporting an association [9–16] and other reporting a lack of association [17–19].

This study is aimed at investigating the relationship between HbAlc and PCE and identifying factors associated with intraoperative and postoperative cardiovascular events in patients with diabetes undergoing complex or moderately complex noncardiac surgery at a university hospital over a 5-year period. The results could provide some help for the clinical and surgical management of such patients.

## 2. Material and Methods

### 2.1. Study Design and Patients

This was a retrospective nested case-control study of patients with diabetes who underwent complex or moderately complex noncardiac surgery at the Second Affiliated Hospital of Xinjiang Medical University from 2013 to 2018. The study was approved by the ethics committee of the Second Affiliated Hospital of Xinjiang Medical University (#XJC201354, approved on March 18, 2014). The requirement for individual consent was waived by the committee.

The inclusion criteria were (1) diagnosis of T2DM, (2) ≥40 years of age, (3) underwent elective noncardiac surgery under general anesthesia, (4) without a pacemaker, (5) HbA1c measurement within 3 months before the operation, and (6) with complete data. The exclusion criteria were (1) emergency operation, (2) intraoperative blood loss ≥ 1200 mL, (3) preoperative hemoglobin ≤ 70 g/L, (4) received a blood transfusion within 90 days before the operation, (5) operation time ≥ 3.8 h and intraoperative blood transfusion ≥ 1 unit, (6) hemodynamic fluctuation ≥ 30%, (7) received small interventional or noninterventional surgery, (8) electrolyte disturbance such as hypokalemia and hypocalcemia during or within 7 days after the operation, or (9) factors that may affect the diagnosis of myocardial ischemia, for example, bundle branch block, preexcitation syndrome, ventricular hypertrophy, drugs inducing ST-T segment changes on ECG, and body position changes that could cause ST-T segment changes on ECG.

### 2.2. Grouping

According to the HbA1c levels at admission, the patients were divided into the HbA1C ≤7%, 7.1%-7.9%, 8.0%-8.9%, and ≥9% groups. This grouping was based on the results of a nested case-control analysis of the ACCORD trial [20].

### 2.3. Data Collection

The general data before the operation were recorded: sex, age, American Society of Anesthesiology (ASA) grade, body mass index (BMI), history of smoking, New York Heart Association (NYHA) grade, course and complications of T2DM, history of hypertension, ECG, cardiac color echocardiography, biochemistry, 2 h postprandial blood glucose, HbA1c, and microalbumin. The occurrence of PCE in the perioperative period (from 30 min before anesthesia to 48 h after the operation) was recorded, including severe ventricular arrhythmia, myocardial ischemia, angina pectoris, nonfatal myocardial infarction, acute heart failure, nonfatal cardiac arrest, and cardiac death.

### 2.4. Anesthesia Method

No drug was used before the operation. Anesthesia induction was performed using an intravenous injection of fentanyl 2-5 *μ*g/kg, propofol 1-2 mg/kg, and vecuronium 0.08-0.10 mg/kg. Mechanical ventilation was given after tracheal intubation, with a tidal volume 5-10 mL/kg and ventilation frequency 12-15 times/min, maintaining PET%CO_2_ at 30-40 mmHg (1 mmHg = 0.133 kPa). Anesthesia was maintained using a target plasma concentration of propofol of 2-5 *μ*g/mL and a target plasma concentration of remifentanil of 2.6 ng/mL. Vecuronium was intermittently injected to maintain muscle relaxation. Sufentanil 5-10 *μ*g was injected intravenously 30 min before the end of the operation. At the end of the operation, the sufentanil was stopped, and intravenous analgesia was used.

### 2.5. Diagnostic Criteria of PCE

(1) Severe ventricular arrhythmia: intraoperative 3-channel dynamic electrocardiogram (DCG) and postoperative ECG showing ventricular extrasystole ≥5 times/min, multifocal or multiform ventricular extrasystole, paired ventricular extrasystole, ventricular tachycardia, R-on-T, atrioventricular block above grade II type 2, and ventricular escape. (2) Myocardial ischemia: postoperative ECG showing obvious ischemic changes compared with before operation. (3) Angina pectoris: clinical symptoms of angina pectoris, and ECG showing ST-segment depression and T-wave inversion. (4) Nonfatal myocardial infarction: related clinical symptoms; DCG showing arrhythmia, mainly ventricular arrhythmia; ECG showing ST-segment elevation with arched upward, new pathological Q-wave appearing with T-wave inversion; blood cTnI concentration > 0.1 ng/mL, blood CK − MB activity > 20 U/L. (5) Acute heart failure: clinical manifestations of pulmonary congestion such as dyspnea, moist rale, and wheezing rale in bilateral lungs, and signs of left or right heart dysfunction such as pulmonary circulation and/or systemic circulation congestion, insufficient tissue blood perfusion. (6) Nonfatal cardiac arrest: sudden ventricular fibrillation or cardiac arrest during operation and rebeating after cardiopulmonary resuscitation, but the patient died within 2 days after the operation.

### 2.6. Statistical Analysis

SPSS 19.0 (IBM, Armonk, NY, USA) was used for statistical analysis. The continuous data are expressed as means ± standard deviations and were analyzed using ANOVA and Tukey's post hoc test. The categorical data are expressed as numbers and percentages and were analyzed using the chi-square test. All variables with *P* values < 0.10 in univariable analyses were included in a multivariable analysis that used PCE as the dependent variable. Two-sided *P* values < 0.05 were considered statistically significant.

## 3. Results

### 3.1. Characteristics of the Patients

**Figure 1:**
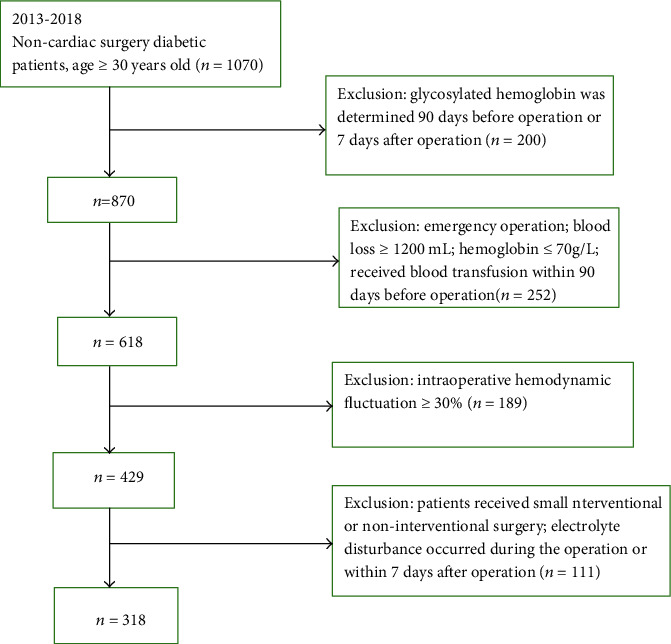
Patient flowchart.

A total of 1070 patients were initially identified; 200 patients were excluded for the lack of HbA1c data within 3 months before operation; 252 were excluded because of emergency surgery, blood loss, low hemoglobin, or blood transfusion; 189 were excluded for hemodynamic fluctuations; and 111 because of the type of surgery (Figure 1). Therefore, 318 patients with a clinical diagnosis of type 2 diabetes, who had complete data and underwent elective noncardiac surgery under general anesthesia, were included in this study. There were 190 males and 128 females. They were 30-78 (62 ± 5.6) years of age. ASA grade was I-III. There were no significant differences in the course of T2DM, high-density lipoprotein cholesterol (HDL-C) levels, and type of operation among the four groups, but there were differences in age, fasting blood glucose, total cholesterol (TC), triglyceride (TG), low-density lipoprotein cholesterol (LDL-C), diastolic blood pressure (DBP), and systolic blood pressure (SBP) (all *P* < 0.001) (Table 1).

### 3.2. Occurrence of PCE

There were 90 cases of PCE among the 318 patients, and the occurrence rate was 28.3%. Among them, the number of patients with myocardial ischemia, serious arrhythmia, serious arrhythmia accompanied by myocardial ischemia, and nonfatal myocardial infarction was 51 (56.7%, 51/90), 31 (34.4%, 31/90), six (6.7%, 6/90), and two (2.2%, 2/90), respectively. No death occurred. The occurrence rate of PCE in the HbA1c 7.1%-7.9% group was 20%. The occurrence rates of PCE in the HbA1c ≥8.0%-8.9% and HbA1c ≥9.0% groups were 30.8% and 35.4%, respectively, and the differences were statistically significant compared with the HbA1c 7.1%-7.9% group (*P* < 0.001). The occurrence rate of PCE in the HbA1c ≤7% group was 25.9%, but the difference was not statistically significant compared with the HbA1c 7.1%-7.9% group (Table 2).

### 3.3. Multivariable Analysis

The course of diabetes, HbA1c stratification, BMI, SBP, DBP, age, and microalbuminuria were associated with PCE in the univariable analyses. The multivariable logistic regression analysis showed that the course of diabetes (HbA1c stratification ≤7%, 7.1%-7.9%, 8.0%-8.9%, ≥9%, OR = 3.672, 95% CI: 1.552-8.687), HbA1c (OR = 1.895, 95% CI: 1.227-4.830), SBP (OR = 1.194, 95% CI: 1.015-2.023), and microalbuminuria (OR = 1.098, 95% CI: 1.005-1.023) were independently associated with PCE in diabetic patients (Table 3).

### 3.4. Occurrence of Diabetic Complications and Comorbidities

Table 4 shows that higher HbA1c levels were associated with coronary heart disease (*P* = 0.02), diabetic nephropathy (*P* = 0.02), and decreased cardiac function (*P* = 0.003). Moreover, with the increase of HbA1c, the cooccurrence of at least two complications increased gradually (*P* = 0.001).

## 4. Discussion

Diabetes has been recognized as an independent risk factor for PCE in patients undergoing cardiac and noncardiac surgeries [7], but the conclusions regarding the association between HbA1c levels and PCE are conflicting [9–19]. Therefore, this study is aimed at investigating the relationship between HbAlc and PCE in patients with diabetes who underwent complex or moderately complex noncardiac surgery. The results strongly suggest that HbA1c levels are related to the incidence of PCE in diabetic patients undergoing complex or moderately complex noncardiac surgery.

There is increasing evidence that diabetes, or even impaired glucose tolerance, can increase the risk of death and perioperative cardiovascular events in patients undergoing noncardiac surgery [7, 15, 21], but there is still a lack of clear conclusion on the effect of different blood glucose control levels on the occurrence of PCE. Blood glucose levels change rapidly within a single day and represent glycemic control at a single time point. Blood glucose levels are associated with perioperative and postoperative complications, especially because anesthesia can increase blood glucose levels [22, 23], but they do not represent the actual long-term glycemic control. On the other hand, HbA1c levels represent the overall glycemic control over the past 3 months and are better indicators of the general diabetes condition in a given patient [1, 24]. Carson et al. [25] conducted a large population-based cohort study to assess the relationship between diabetes and HbA1c and the risk of intracerebral hemorrhage (ICH), and the results showed that HbA1c had a nonlinear J-shaped relationship with ICH in diabetic patients (nonlinear *P* = 0.0186). Compared with the fourth HbA1c decile of 6.5%-6.7%, the HR of ICH in patients with the lowest HbA1c decile ≤ 6.0% and the highest HbA1c decile > 9.3% (>78 mmol/mol) was 1.27 and 2.19, respectively. The conclusion was that the increased risk of diabetes-related ICH was directly related to the course of diabetes. The relationship between intracerebral hemorrhage and HbA1c appears to have a “J” shape, indicating that both poor and extremely intensive diabetes control might be associated with an increased risk of stroke. Kotfis et al. [26] showed that elevated HbA1c levels are associated with postoperative delirium regardless of the diagnosis of diabetes. Those results are supported by previous studies [9–16] but contradict others [17–19]. Differences in patient characteristics, ethnicity, and genetics might be involved in the discrepancies.

Previous studies found that emergency operation, operation time ≥ 3.8 h, intraoperative blood transfusion ≥ 1 unit, and hemodynamic fluctuation ≥ 30% were independent risk factors of PCE [8, 15, 21, 25, 27]. Therefore, in order to eliminate those confounding factors, patients fitting those criteria were excluded. The results showed that the incidence of PCE was as high as 30.8% in patients with the worst glucose control (HbA1c ≥ 9%) compared with those with HbA1c 7.0%-7.9%. Meanwhile, although nonsignificant, the patients with HbA1c < 7.0% showed a slightly higher PCE rate than the HbA1c 7.0%-7.9%, suggesting a J-shaped curve.

Myocardial ischemia was the most common PCE in diabetic patients in the present study, as supported by previous studies [6, 28, 29]. Severe myocardial ischemia immediately causes symptoms of angina pectoris and can lead to dismal outcomes. One of the vascular complications in diabetic patients is intradermal cell proliferation of the vascular wall and subendothelial fibrosis with elastic fiber proliferation, leading to coronary artery lumen contraction [30]. Glycated metabolites could increase the secretion of vasoconstrictor factors while decreasing the secretion of endogenous vasodilator factors such as NO [25]. In addition, the disturbance of the cardiac autonomic nerve increases the oxygen consumption of the myocardium, further aggravating myocardial ischemia [31].

The patients in the highest HbA1c group were older and had higher cardiovascular risk factors, including higher LDL-C, TG, TC, SBP, and DBP, which is supported by the literature [32, 33]. The high levels of HbA1c represent the poor control of blood glucose over the past 3 months [1, 24]. The hyperglycemia in diabetic patients damages the vascular endothelial cells, increases platelet activity, and abnormal lipid and protein metabolism increases the coagulation state and viscosity, and increases the oxygen free radicals, leading to microcirculation issues and tissue hypoxia, promoting atherosclerosis of large blood vessels such as coronary artery and cerebral vessels, and elevating the blood pressure [34]. Lipid metabolism disturbances are also related to insulin resistance [35].

This study is the first to assess the relationship between blood glucose control and PCE in patients who underwent noncardiac surgery, but there are still some limitations to this study. First, this is a single-center study. Second, many patients who met the diagnostic criteria were excluded because of the lack of HbA1c data, resulting in a small sample size. Third, even if most patients had HbA1c measured on the day of admission, HbA1c was measured 2-3 months before the operation in some patients. Fourth, the incidence of hypoglycemia was not analyzed, and the factors of some antiatherosclerotic drugs on PCE were not evaluated. Therefore, further research is needed to determine the recommended objective of blood glucose control.

## 5. Conclusions

HbA1c levels are related to the incidence of PCE in diabetic patients undergoing complex or moderately complex noncardiac surgery.

## Figures and Tables

**Table 1:** Comparison of the characteristics of the subjects among the four groups.

	HbA1c ≤ 7.0%	HbA1c 7.1%-7.9%	HbA1c 8.0%-8.9%	HbA1c ≥ 9%	*P*
	*n* = 77	*n* = 110	*n* = 80	*n* = 50	
Age (years)	52.2 ± 6.0	51.8 ± 6.6	54.8 ± 6.1	56.8 ± 6.8	<0.001
Course of disease (years)	6.5 ± 2.5	6.6 ± 1.8	6.6 ± 2.1	6.6 ± 2.3	0.06
FPG (mmol/L)	6.86 ± 0.94	7.28 ± 1.10	7.99 ± 1.20	11.8 ± 1.8	<0.001
TG (mmol/L)	1.49 ± 0.60	1.56 ± 0.61	1.67 ± 0.76	1.80 ± 0.77	<0.001
TC (mmol/L)	4.60 ± 0.80	4.68 ± 0.80	4.78 ± 0.88	4.93 ± 0.87	<0.001
LDL-C (mmol/L)	0.99 ± 0.19	1.01 ± 0.24	1.03 ± 0.28	1.07 ± 0.27	<0.001
HDL-C (mmol/L)	1.27 ± 0.28	1.18 ± 0.26	1.15 ± 0.34	1.15 ± 0.31	0.07
SBP (mmHg)	136 ± 14	137 ± 0.14	142 ± 18	143 ± 16	<0.001
DBP (mmHg)	74 ± 7	75 ± 6	76 ± 5	79 ± 8	<0.001
Type of operation					
Abdomen	15 (19.4%)	25 (22.7%)	16 (20%)	11 (22%)	0.91
Chest	16 (20.7%)	22 (20%)	18 (22.5%)	9 (18%)	0.91
Bone	20 (25.9%)	25 (22.7%)	17 (21.2%)	12 (24%)	0.71
Urinary system	12 (15.6%)	17 (15.4%)	17 (21.2%)	9 (18%)	0.24
Others	14 (18.1)	21 (19.0%)	12 (15%)	9 (18%)	0.92

HbA1c: glycated hemoglobin; FPG: fasting plasma glucose; TG: triglyceride; TC: total cholesterol; LDL-C: low-density lipoprotein cholesterol; HDL-C: high-density lipoprotein cholesterol; SBP: systolic blood pressure; DBP: diastolic blood pressure.

**Table 2:** Perioperative cardiovascular events among the HbA1c groups.

Events	HbA1c ≤ 7%	HbA1c 7.1-7.9%	HbA1c 8.0-8.9%	HbA1c ≥ 9%
	*n* = 77	*n* = 110	*n* = 80	*n* = 51
Yes	20 (25.9%)	22 (20%)	25 (30.8%)	18 (35.3%)
No	55 (74.1%)	88 (80%)	55 (69.2%)	33 (64.7%)

Events: perioperative cardiovascular event; HbA1c: glycated hemoglobin.

**Table 3:** Multivariable and unconditional logistic regression analysis of the factors associated with perioperative cardiovascular events.

	OR	95% confidence interval of OR		*P*
		Lower limit	Higher limit	
Long course of disease	3.672	1.552	8.687	0.003
HbA1c	1.895	1.227	4.830	0.001
SBP	1.195	1.015	2.023	0.006
Microalbuminuria	1.098	1.005	1.023	0.017

OR: odds ratio; HbA1c: glycated hemoglobin; SBP: systolic blood pressure.

**Table 4:** Comparison of the occurrence of diabetic complications and comorbidities among subjects in the four groups.

	HbA1c ≤ 7%	HbA1c 7.1-7.9%	HbA1c 8.0-8.9%	HbA1c ≥ 9%	*P*
	*n* = 77	*n* = 110	*n* = 25	*n* = 18	
Hypertension	28%	26%	30%	29%	0.25
Coronary heart disease	41%	32%	36%	46%	0.02
Nephropathy	1.8%	2.8%	2.2%	3.7%	0.02
Cerebrovascular disease	4.4%	3.9%	4.1%	4.5%	0.22
EF < 50%	3.9%	6.4%	6.3%	6.9%	0.003
≥2 complications	14.2%	15.4%	15.8%	21.8%	0.001

HbA1c: glycated hemoglobin; EF: ejection fraction.

## Data Availability

The data used to support the findings of this study are available from the corresponding author upon request.

## References

[1] (2019). Introduction: standards of medical care in diabetes—2020. *Diabetes Care*.

[2] NCDRF Collaboration (2016). Worldwide trends in diabetes since 1980: a pooled analysis of 751 population-based studies with 4.4 million participants. *The Lancet*.

[3] Xu G., Liu B., Sun Y. (2018). Prevalence of diagnosed type 1 and type 2 diabetes among US adults in 2016 and 2017: population based study. *BMJ*.

[4] American Diabetes Association (2019). 8. Obesity management for the treatment of type 2 diabetes: standards of medical care in diabetes-2019. *Diabetes Care*.

[5] Qaseem A., Barry M. J., Humphrey L. L., Forciea M. A., for the Clinical Guidelines Committee of the American College of Physicians (2017). Oral pharmacologic treatment of type 2 diabetes mellitus: a clinical practice guideline update from the American College of Physicians. *Annals of Internal Medicine*.

[6] Yeh C. C., Liao C. C., Chang Y. C. (2013). Adverse outcomes after noncardiac surgery in patients with diabetes: a nationwide population-based retrospective cohort study. *Diabetes Care*.

[7] Biteker M., Dayan A., Can M. M. (2011). Impaired fasting glucose is associated with increased perioperative cardiovascular event rates in patients undergoing major non-cardiothoracic surgery. *Cardiovascular Diabetology*.

[8] Andersson C., van Gaal L., Caterson I. D. (2012). Relationship between HbA1c levels and risk of cardiovascular adverse outcomes and all-cause mortality in overweight and obese cardiovascular high-risk women and men with type 2 diabetes. *Diabetologia*.

[9] Ouattara A., Lecomte P., Le Manach Y. (2005). Poor intraoperative blood glucose control is associated with a worsened hospital outcome after cardiac surgery in diabetic patients. *Anesthesiology*.

[10] Latham R., Lancaster A. D., Covington J. F., Pirolo J. S., Thomas C. S. (2001). The association of diabetes and glucose control with surgical-site infections among cardiothoracic surgery patients. *Infection Control and Hospital Epidemiology*.

[11] Lazar H. L., Chipkin S. R., Fitzgerald C. A., Bao Y., Cabral H., Apstein C. S. (2004). Tight glycemic control in diabetic coronary artery bypass graft patients improves perioperative outcomes and decreases recurrent ischemic events. *Circulation*.

[12] Halkos M. E., Puskas J. D., Lattouf O. M. (2008). Elevated preoperative hemoglobin A1c level is predictive of adverse events after coronary artery bypass surgery. *The Journal of Thoracic and Cardiovascular Surgery*.

[13] Cohen O., Dankner R., Chetrit A. (2003). Multidisciplinary intervention for control of diabetes in patients undergoing coronary artery bypass graft (CABG). *Cardiovascular Surgery*.

[14] Iavazzo C., McComiskey M., Datta M. (2016). Preoperative HBA1c and risk of postoperative complications in patients with gynaecological cancer. *Archives of Gynecology and Obstetrics*.

[15] Jehan F., Khan M., Sakran J. V. (2018). Perioperative glycemic control and postoperative complications in patients undergoing emergency general surgery. *The Journal of Trauma and Acute Care Surgery*.

[16] Yong P. H., Weinberg L., Torkamani N. (2018). The presence of diabetes and higher HbA1c are independently associated with adverse outcomes after surgery. *Diabetes Care*.

[17] Aydinli B., Demir A., Ozmen H., Vezir O., Unal U., Ozdemir M. (2018). Can pre-operative HbA1c values in coronary surgery be a predictor of mortality?. *Turkish Journal of Anaesthesiology and Reanimation*.

[18] Singh N., Zeng C., Lewinger J. P. (2019). Preoperative hemoglobin A1c levels and increased risk of adverse limb events in diabetic patients undergoing infrainguinal lower extremity bypass surgery in the Vascular Quality Initiative. *Journal of Vascular Surgery*.

[19] Kranenburg G., van der Graaf Y., van der Leeuw J. (2015). The relation between HbA1c and cardiovascular events in patients with type 2 diabetes with and without vascular disease. *Diabetes Care*.

[20] Colayco D. C., Niu F., McCombs J. S., Cheetham T. C. (2011). A1C and cardiovascular outcomes in type 2 diabetes: a nested case-control study. *Diabetes Care*.

[21] Ogurtsova K., da Rocha Fernandes J. D., Huang Y. (2017). IDF Diabetes Atlas: global estimates for the prevalence of diabetes for 2015 and 2040. *Diabetes Research and Clinical Practice*.

[22] Wang J., Chen K., Li X. (2019). Postoperative adverse events in patients with diabetes undergoing orthopedic and general surgery. *Medicine*.

[23] Duggan E. W., Carlson K., Umpierrez G. E. (2017). Perioperative hyperglycemia management: an update. *Anesthesiology*.

[24] Krhac M., Lovrencic M. V. (2019). Update on biomarkers of glycemic control. *World Journal of Diabetes*.

[25] Carson J. L., Scholz P. M., Chen A. Y., Peterson E. D., Gold J., Schneider S. H. (2002). Diabetes mellitus increases short-term mortality and morbidity in patients undergoing coronary artery bypass graft surgery. *Journal of the American College of Cardiology*.

[26] Kotfis K., Szylinska A., Listewnik M., Brykczynski M., Ely E. W., Rotter I. (2019). Diabetes and elevated preoperative HbA1c level as risk factors for postoperative delirium after cardiac surgery: an observational cohort study. *Neuropsychiatric Disease and Treatment*.

[27] Thourani V. H., Weintraub W. S., Stein B. (1999). Influence of diabetes mellitus on early and late outcome after coronary artery bypass grafting. *The Annals of Thoracic Surgery*.

[28] Newman J. D., Wilcox T., Smilowitz N. R., Berger J. S. (2018). Influence of diabetes on trends in perioperative cardiovascular events. *Diabetes Care*.

[29] Sari M., Kilic H., Ariturk O. K., Yazihan N., Akdemir R. (2015). Diabetic patients have increased perioperative cardiac risk in heart-type fatty acid-binding protein-based assessment. *Medical Principles and Practice : International Journal of the Kuwait University, Health Science Centre*.

[30] Einarson T. R., Acs A., Ludwig C., Panton U. H. (2018). Prevalence of cardiovascular disease in type 2 diabetes: a systematic literature review of scientific evidence from across the world in 2007-2017. *Cardiovascular Diabetology*.

[31] Liu Q., Chen D., Wang Y., Zhao X., Zheng Y. (2012). Cardiac autonomic nerve distribution and arrhythmia. *Neural Regeneration Research*.

[32] Little S. A., Jarnagin W. R., DeMatteo R. P., Blumgart L. H., Fong Y. (2002). Diabetes is associated with increased perioperative mortality but equivalent long-term outcome after hepatic resection for colorectal cancer. *Journal of gastrointestinal surgery*.

[33] Hughes K., Jackson J. D., Prendergast T. I. (2013). Diabetes mellitus is not associated with major morbidity following open abdominal aortic aneurysm repair. *The Journal of Surgical Research*.

[34] Ata A., Valerian B. T., Lee E. C., Bestle S. L., Elmendorf S. L., Stain S. C. (2010). The effect of diabetes mellitus on surgical site infections after colorectal and noncolorectal general surgical operations. *The American Surgeon*.

[35] Krolikowska M., Kataja M., Poyhia R., Drzewoski J., Hynynen M. (2009). Mortality in diabetic patients undergoing non-cardiac surgery: a 7-year follow-up study. *Acta Anaesthesiologica Scandinavica*.

